# Living alone, long-term cardiometabolic stability, and medication management in adults with type 2 diabetes: a secondary analysis of the ACCORD trial

**DOI:** 10.3389/fendo.2026.1906084

**Published:** 2026-07-31

**Authors:** Yanhui Chen, Zhenhua Xing, Jiaqing Hu

**Affiliations:** 1 The Second People’s Hospital of Hunan Province (Brain Hospital of Hunan Province), Changsha, Hunan, China; 2 Department of Emergency Medicine, Second Xiangya Hospital, Central South University, Changsha, Hunan, China

**Keywords:** blood pressure variability, glycemic variability, living alone, medication adherence, neuropathy, type 2 diabetes

## Abstract

**Background:**

Living alone is an easily ascertained household characteristic that may shape diabetes self-management. Its relation to long-term cardiometabolic stability and medication-management patterns remains unclear.

**Methods:**

We conducted a secondary analysis of the ACCORD trial. After excluding two participants with missing baseline adult co-residence information, living alone was defined as not living with another adult at baseline. Outcomes were repeated HbA1c, fasting plasma glucose (FPG), systolic blood pressure (SBP), diastolic blood pressure (DBP), low-density lipoprotein cholesterol (LDL), and high-density lipoprotein cholesterol (HDL). Mixed-effects models estimated differences in repeated levels, linear models estimated within-person variability (standard deviation and average real variability), and exploratory Cox models examined microvascular and neuropathy outcomes. Medication adherence and treatment-management patterns were assessed from longitudinal ACCORD records.

**Results:**

Among 10,249 participants, 2,078 (20.3%) were classified as living alone. Participants living alone were older, more often female, and had more baseline depression. In fully adjusted mixed-effects models, living alone was associated with slightly higher HbA1c (beta 0.06%, 95% CI 0.03 to 0.10), FPG (beta 2.28 mg/dL, 95% CI 0.72 to 3.83), DBP (beta 0.40 mmHg, 95% CI 0.05 to 0.75), and LDL cholesterol (beta 1.77 mg/dL, 95% CI 0.45 to 3.09). Living alone was also associated with greater variability, most consistently for SBP and DBP; HbA1c SD and HDL SD/ARV remained below family-specific false-discovery-rate thresholds, whereas HbA1c ARV was borderline. The DBP mean-level association was attenuated when analyses began at month 12, and neuropathy outcomes were retained as exploratory. Suboptimal oral glucose-lowering medication adherence was descriptively more frequent among participants living alone, but the adjusted association was borderline (adjusted OR 1.08, 95% CI 1.00 to 1.17; P = 0.052).

**Conclusions:**

In ACCORD, living alone was associated with small differences in glycemic and lipid levels, greater variability in selected cardiometabolic indicators, and medication-management patterns linked to subsequent metabolic control. Findings suggest self-management instability rather than a uniform increase in vascular outcomes.

## Introduction

Adults with type 2 diabetes remain at high risk of cardiovascular and microvascular complications despite advances in glucose-, blood-pressure-, and lipid-lowering therapy (1). Traditional risk-factor control is central to diabetes management, yet multifactorial intervention does not fully eliminate excess vascular risk (2). Part of this residual risk is likely to arise from social and behavioral conditions that affect what happens between clinic visits, including household structure, social support, and the capacity to carry out daily self-management (3).

Loneliness, social isolation, and living arrangements capture related but distinct dimensions of social context. A recent UK Biobank analysis reported that among adults with diabetes, loneliness, but not a composite social-isolation index, was associated with incident cardiovascular disease and showed additive interaction with traditional risk-factor control (4). In a prior ACCORD analysis, living alone was not associated with cardiovascular events or hypoglycemia (5). That study addressed hard clinical endpoints, whereas the present analysis focuses on longitudinal cardiometabolic levels, visit-to-visit variability, and medication-management patterns. Living alone is not equivalent to loneliness, social isolation, or lack of social support. In this study, it was treated as an objective household characteristic that may mark the absence of an adult co-resident who could assist with medication routines, recognition of symptoms, or responses to treatment recommendations (6).

Prior studies have focused mainly on hard cardiovascular endpoints or cross-sectional metabolic measurements (7). This leaves two practical questions for diabetes care: whether living alone is related to long-term trajectories and visit-to-visit variability, and whether medication-management behaviors help explain any differences. Mean risk-factor levels do not fully describe self-management stability; two patients with similar average HbA1c, blood pressure, or lipid values can differ substantially in variability over time (8, 9). Medication adherence provides a behavioral link, and longitudinal studies have shown that poorer diabetes medication adherence is associated with worse glycemic control (10).

Using data from the ACCORD trial, we examined repeated protocolized measurements of glycemia, blood pressure, and lipids together with longitudinal medication records in more than 10,000 adults with type 2 diabetes at high cardiovascular risk. We evaluated whether living alone was associated with: (1) longitudinal levels of HbA1c, FPG, SBP, DBP, LDL, and HDL; (2) within-person variability in these six indicators; (3) medication adherence and treatment-management patterns; and (4) exploratory microvascular and neuropathy outcomes. We expected living alone to be associated more clearly with cardiometabolic instability and medication-management patterns than with large absolute differences in mean risk-factor levels.

## Methods

### Study design and population

This was a secondary analysis of the ACCORD trial. ACCORD enrolled 10,251 adults aged 40 to 79 years with type 2 diabetes, HbA1c >=7.5%, and either prior clinical cardiovascular disease or, among those without prior cardiovascular disease, additional cardiovascular risk factors (11, 12). All participants were randomized to intensive versus standard glycemic therapy. Within the glycemia trial, participants were also assigned either to a blood-pressure trial comparing intensive versus standard SBP targets (n=4,733) or to a lipid trial comparing fenofibrate versus placebo on a background of open-label simvastatin therapy (n=5,518) (11, 13).

Of 10,251 randomized participants, 2 were excluded because baseline adult co-residence information was missing, leaving an analytic cohort of 10,249 participants with covariates required for the primary models. Of these, 8,171 (79.7%) reported living with one or more adults and 2,078 (20.3%) reported not living with another adult. The ACCORD baseline variable available for this analysis did not separately identify participants living with children or other dependents but no adults, nor did it provide information on changes in household composition during follow-up. Because ACCORD measurements and treatment records were stored in domain-specific datasets, sample sizes varied across analyses according to the availability of repeated metabolic measurements, medication-management records, and microvascular outcome data. Blood-pressure medication analyses were restricted to participants in the blood-pressure trial, and lipid medication analyses were restricted to participants in the lipid trial. Definitions of key exposure, medication, and outcome variables are provided in **Supplementary Table 1**, and available baseline living-status categories are shown in **Supplementary Table 2**.

### Exposure and metabolic outcomes

The primary exposure was baseline adult co-residence status. Participants were classified as living alone if the ACCORD baseline household-composition item indicated that they did not live with another adult; the reference group consisted of participants who lived with one or more adults. Thus, the exposure should be interpreted as absence of an adult co-resident rather than a comprehensive measure of household size, marital status, loneliness, or social support. Participants living with children or dependents but no other adults could not be distinguished from those living entirely alone in the available ACCORD data. Living-alone status was assessed once at baseline and was not updated during follow-up. The assumed relations among household context, measured and unmeasured social or clinical factors, medication routines, cardiometabolic stability, and exploratory complications are summarized in **Supplementary Figure 1**.

**Figure 1:**
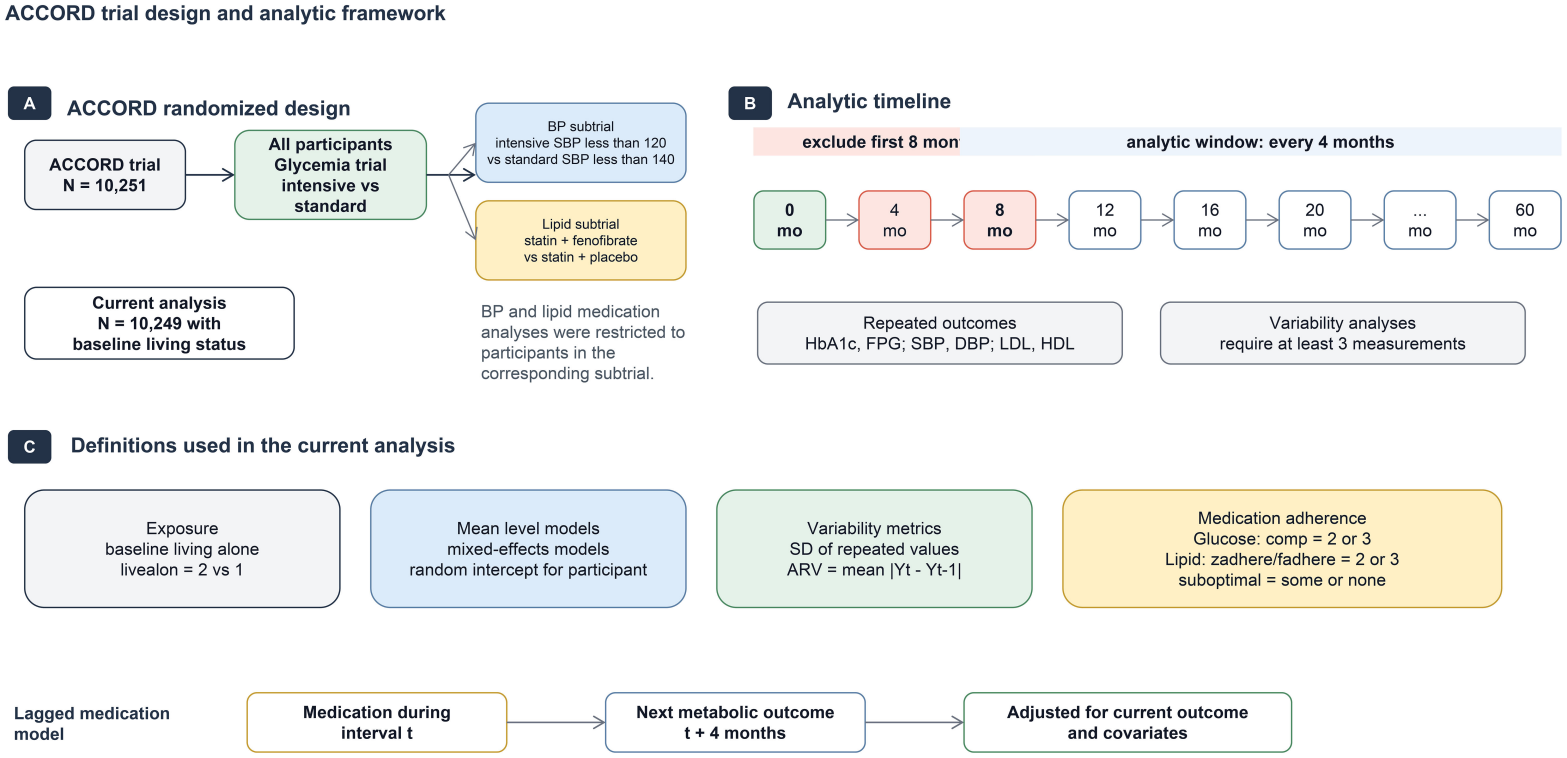
ACCORD randomized design and analytic framework. **(A)** ACCORD randomized treatment design and derivation of the current analytic cohort. **(B)** Analytic timeline and repeated-measurement window. **(C)** Definitions of the exposure, repeated metabolic outcomes, variability measures, and medication-management variables. The lower pathway illustrates the lagged medication-outcome analysis.

The primary repeated metabolic outcomes were HbA1c (%), FPG (mg/dL), SBP (mmHg), DBP (mmHg), LDL cholesterol (mg/dL), and HDL cholesterol (mg/dL). The ACCORD randomized design, early treatment-intensification period, and analytic timeline are summarized in **Figure 1**. The first months after randomization involved rapid protocol-driven treatment changes; therefore, the primary post-stabilization analyses excluded early follow-up and used measurements from month 8 onward. We also conducted sensitivity analyses using all randomized visits and alternative analytic start points at months 4, 8, and 12.

For each metabolic indicator, within-person variability was measured using standard deviation (SD) and average real variability (ARV). ARV was calculated as the mean absolute difference between consecutive non-missing post-stabilization visits. Participants were required to have at least three non-missing measurements for each variability analysis.

### Medication adherence and treatment management

Medication-management analyses used the ACCORD glucose-medication logs, blood-pressure trial medication records, and lipid trial medication-management records. These visit-based records captured prescribed medications, medication class or dose, medication action or modification codes, reason codes for medication changes or no change, and participant-estimated adherence at protocol visits. Derived medication variables included oral glucose-medication class count and adherence, glucose-therapy actions, blood-pressure treatment changes and no-change reasons, simvastatin and fenofibrate/placebo adherence, simvastatin dose or nonuse, and other lipid-lowering medication use. Suboptimal adherence was defined as some of the time or none of the time. A stricter sensitivity definition classified only none of the time as suboptimal adherence. Detailed coding definitions are provided in **Supplementary Table 1**.

### Exploratory microvascular and neuropathy outcomes

Exploratory time-to-event analyses examined ACCORD microvascular outcomes using the published ACCORD microvascular outcome framework (14). Composite outcomes included a UKPDS-style microvascular composite of advanced nephropathy or retinal procedure and an expanded composite that also included MNSI-defined neuropathy. Individual outcomes covered nephropathy, retinopathy, and neuropathy domains, including albuminuria, renal failure or ESRD/dialysis, retinal photocoagulation or vitrectomy, visual acuity decline, MNSI score >2.0, vibratory sensation loss, ankle jerk loss, and pressure sensation loss. Detailed definitions are listed in **Supplementary Table 1**.

### Statistical analysis

Baseline characteristics were summarized as mean (SD) for approximately normally distributed continuous variables, median (interquartile range) for skewed continuous variables, and counts with percentages for categorical variables. Mixed-effects models with participant-specific random intercepts were used to estimate the association between living alone and repeated post-stabilization metabolic levels. Three adjustment models were specified: Model 1 included living-alone status only; Model 2 adjusted for age, sex, race, education, and domain-specific randomized treatment arm(s); and Model 3 further adjusted for BMI, diabetes duration, baseline cardiovascular disease, heart failure, depression, smoking, and alcohol use. Relevant randomized treatment arms were defined by outcome domain: glycemia arm for HbA1c and FPG, glycemia plus blood-pressure arm for SBP and DBP, and glycemia plus lipid arm for LDL and HDL. The fully adjusted model was treated as the primary model. We also ran a same-sample depression sensitivity analysis in which baseline depression was added as the only incremental covariate to the otherwise fully adjusted model without depression.

Additional measured covariates used in sensitivity analyses were defined from ACCORD public-use files after a covariate audit. Baseline insurance coverage was derived from the ins_cover and dk_unins items and categorized as insured versus uninsured or unknown. Prescription drug-benefit status was taken from the baseline drugbene item, which was retained using the ACCORD CRF four-category coding (Yes, No, Don’t know, Uninsured) and was not recoded to match the separately derived any-insurance variable. Baseline cognitive function was assessed using the TOTAL_MMSE score, which was available only in the ACCORD-MIND subset; MMSE-adjusted models were therefore treated as subset sensitivity analyses rather than full-cohort primary models. These additional covariates were not included in the primary Model 3 specification but were added in sensitivity analyses to evaluate residual confounding; tabulated baseline distributions for the interpretable measured covariates are shown in **Supplementary Table 3**.

Linear regression models evaluated the association between living alone and variability metrics; fully adjusted variability models included the covariates in Model 3 and the participant’s mean level of the corresponding metabolic indicator. Medication-management outcomes were analyzed using linear or logistic models, as appropriate, adjusted for the fully adjusted covariate set, the corresponding domain-specific randomized treatment arm(s), and visit month when applicable, with participant-clustered robust standard errors.

Lagged 4-month interval models evaluated whether medication-management exposures during each interval were associated with the next metabolic measurement, adjusting for the current value of the same metabolic outcome, living-alone status, visit interval, demographic factors, the corresponding domain-specific randomized treatment arm(s), clinical covariates, and health behaviors; robust standard errors were clustered by participant. For adherence exposures, intervals with no non-missing adherence information were excluded from the corresponding model. These lagged models established temporal ordering but did not eliminate time-varying confounding; treatment intensification can reflect clinical response to worse metabolic control rather than an adverse effect of treatment.

Cox proportional hazards models estimated hazard ratios for exploratory microvascular and neuropathy outcomes, adjusting for the fully adjusted covariate set. Continuous outcomes in mixed-effects and linear models are reported as beta coefficients with 95% confidence intervals. Binary medication-management outcomes are reported as odds ratios with 95% confidence intervals. Time-to-event outcomes are reported as hazard ratios with 95% confidence intervals. Benjamini-Hochberg false-discovery-rate sensitivity analyses were conducted from raw model P values within the longitudinal metabolic-level, metabolic-variability, and exploratory microvascular/neuropathy outcome families; a broader correction across all reported outcome tests was also tabulated as a sensitivity check. Findings that did not remain below the false-discovery-rate threshold were interpreted as exploratory. Analyses were conducted using Stata/MP 17.0 (StataCorp LLC, College Station, TX, USA) and R version 4.3.0 (R Foundation for Statistical Computing, Vienna, Austria). Two-sided P values less than 0.05 were considered statistically significant for nominal analyses.

## Results

### Baseline characteristics

Among 10,249 participants, 2,078 (20.3%) did not live with another adult at baseline and 8,171 (79.7%) lived with one or more adults (**Table 1**). Participants not living with another adult were slightly older (median 63 vs 62 years), more often female (53.0% vs 34.9%), and less often White (58.5% vs 63.4%). Baseline depression was more common among participants not living with another adult (29.9% vs 22.0%). Baseline HbA1c and FPG were similar between groups, whereas LDL and HDL cholesterol were slightly higher among participants not living with another adult.

**Table 1:** Baseline characteristics of ACCORD participants by living alone status.

Variables	Overall	Living alone	Not living alone	p-value
N	10,249	2,078	8,171	
Demographics				
Age (yrs)	62.0 (58.0–67.0)	63.0 (58.0–68.0)	62.0 (58.0–67.0)	<0.001
Female	38.5% (3,950)	53.0% (1,102)	34.9% (2,848)	<0.001
White race	62.4% (6,392)	58.5% (1,215)	63.4% (5,177)	<0.001
Education				0.95
Less than high school	14.8% (1,521)	14.5% (302)	14.9% (1,219)	
High-school graduate	26.4% (2,704)	25.8% (535)	26.6% (2,169)	
Some college	32.8% (3,357)	33.9% (704)	32.5% (2,653)	
College degree or higher	26.0% (2,661)	25.8% (535)	26.0% (2,126)	
Diabetes and metabolic parameters				
Duration of diabetes (yrs)	10.0 (5.0–15.0)	9.0 (5.0–15.0)	10.0 (5.0–15.0)	1.00
BMI (kg/m²)	31.8 (28.2–35.9)	32.3 (28.5–36.6)	31.7 (28.1–35.7)	<0.001
WC(cm)	106.7 (96.5–116.0)	106.7 (97.0–116.8)	106.7 (96.5–116.0)	0.81
HbA1c (%)	8.1 (7.6–8.9)	8.2 (7.6–8.9)	8.1 (7.6–8.8)	0.051
FPG (mg/dL)	167.0 (138.0–204.0)	169.0 (139.0–206.0)	167.0 (138.0–204.0)	0.25
Current smoker	13.9% (1,429)	15.5% (323)	13.5% (1,106)	0.062
Alcohol consumption (drinks/week)	0.0 (0.0–0.0)	0.0 (0.0–0.0)	0.0 (0.0–0.0)	<0.001
CVD history	35.2% (3,608)	32.1% (666)	36.0% (2,942)	0.003
Neuropathy	26.7% (2,736)	28.4% (591)	26.3% (2,145)	0.13
Depression	23.6% (2,419)	29.9% (622)	22.0% (1,797)	<0.001
Eye disease	31.2% (3,193)	34.3% (712)	30.4% (2,481)	0.003
Heart failure	4.8% (494)	5.2% (108)	4.7% (386)	0.67
Proteinuria	19.9% (2,035)	20.6% (428)	19.7% (1,607)	0.64
Blood pressure and lipids				
Systolic BP (mmHg)	135.0 (125.0–147.0)	135.0 (124.0–147.0)	135.0 (125.0–147.0)	0.99
Diastolic BP (mmHg)	75.0 (68.0–82.0)	74.0 (67.0–82.0)	75.0 (68.0–82.0)	0.80
LDL cholesterol (mg/dL)	101.0 (81.0–125.0)	103.0 (82.0–128.0)	100.0 (81.0–124.0)	0.009
HDL cholesterol (mg/dL)	40.0 (34.0–48.0)	41.0 (35.0–50.0)	39.0 (34.0–47.0)	<0.001
Medications				
Metformin (biguanides)	63.9% (6,554)	61.4% (1,276)	64.6% (5,277)	0.026
Sulfonylureas	53.4% (5,474)	51.6% (1,073)	53.9% (4,400)	0.19
Thiazolidinediones	22.0% (2,258)	18.9% (392)	22.8% (1,866)	<0.001
Any insulin	34.9% (3,582)	36.8% (765)	34.5% (2,816)	0.13
Statins	63.7% (6,500)	61.1% (1,261)	64.3% (5,238)	0.028
ACE inhibitors	54.5% (5,568)	54.4% (1,126)	54.5% (4,442)	1.00
ARBs	16.7% (1,712)	16.4% (340)	16.8% (1,370)	0.92
Beta-blockers	30.1% (3,079)	28.9% (598)	30.4% (2,481)	0.40
Aspirin	54.7% (5,579)	54.5% (1,123)	54.7% (4,456)	0.98
Fibrate	6.15%(627)	5.19% (107)	6.39% (520)	0.04
CCB	11.67%(1,193)	13.15% (272)	11.30%(921)	0.02

Data are presented as median (interquartile range) for continuous variables and % (n) for categorical variables. P-values were calculated using the Wilcoxon rank-sum test for continuous variables and the chi-square test for categorical variables. The diabetes-duration p-value reflects the comparison of the full rank distribution; rounded medians and interquartile ranges may appear different even when the rank-sum test indicates no meaningful distributional difference.

ACE, angiotensin-converting enzyme; ARB, angiotensin II receptor blocker; BMI, body mass index; BP, blood pressure; CCB, calcium channel blocker; CVD, cardiovascular disease; FPG, fasting plasma glucose; HbA1c, glycated hemoglobin; HDL, high-density lipoprotein; LDL, low-density lipoprotein; WC, waist circumference.

### Longitudinal metabolic levels

**Figure 2:**
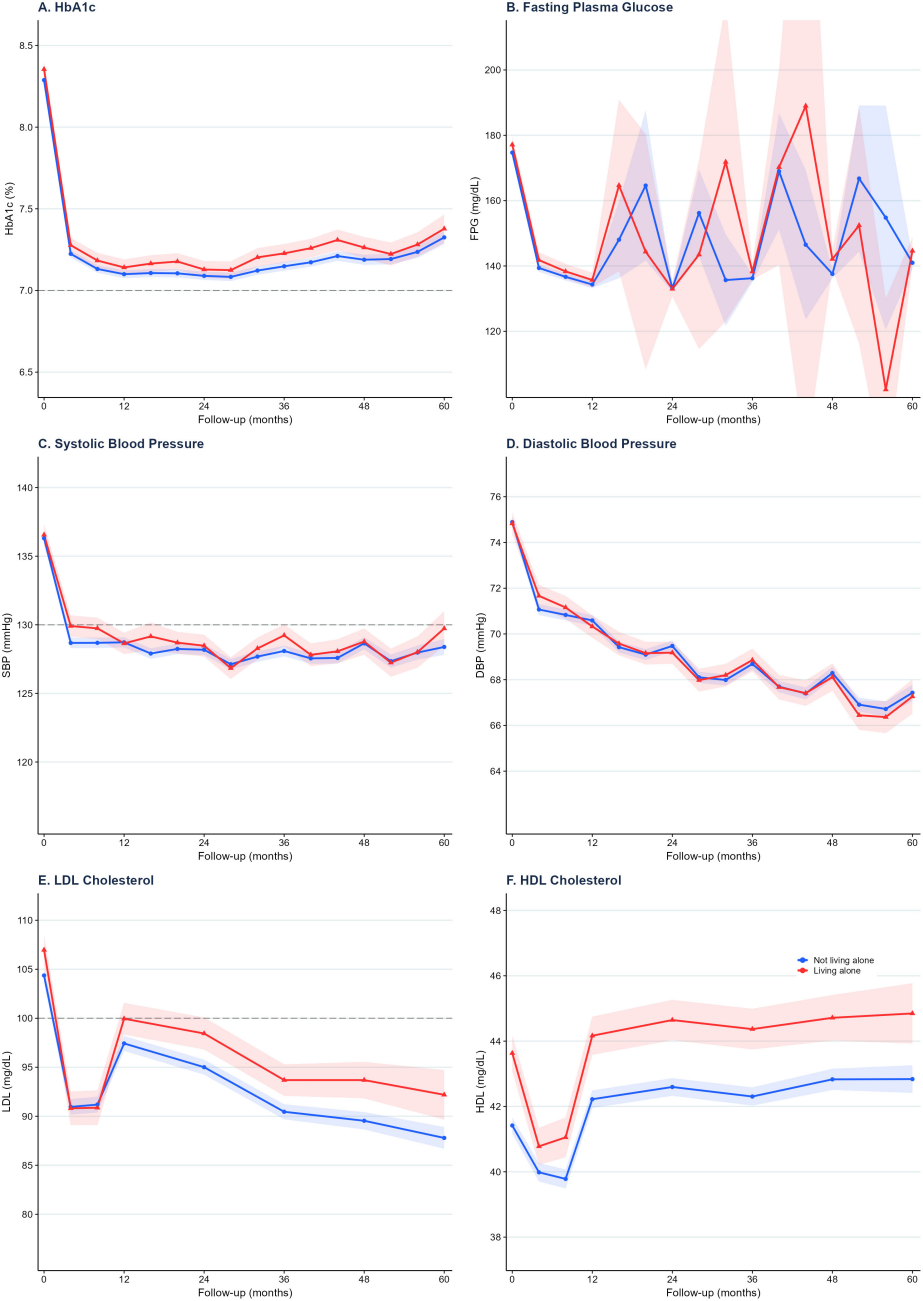
Longitudinal trajectories of metabolic indicators by living-alone status. **(A)** HbA1c, **(B)** fasting plasma glucose, **(C)** systolic blood pressure, **(D)** diastolic blood pressure, **(E)** LDL cholesterol, and **(F)** HDL cholesterol. Points indicate mean values, and shaded bands indicate 95% confidence intervals.

Mean trajectories of the six metabolic indicators over time are shown in **Figure 2**. In fully adjusted mixed-effects models (**Table 2**; **Supplementary Figure 2**), living alone was associated with small differences toward higher HbA1c (beta 0.06%, 95% CI 0.03 to 0.10; P = 0.001), higher FPG (beta 2.28 mg/dL, 95% CI 0.72 to 3.83; P = 0.004), higher DBP (beta 0.40 mmHg, 95% CI 0.05 to 0.75; P = 0.027), and higher LDL cholesterol (beta 1.77 mg/dL, 95% CI 0.45 to 3.09; P = 0.009). Living alone was not significantly associated with SBP or HDL after full adjustment. Complete-case fully adjusted model sample sizes ranged from 44,732 to 141,040 observations across 9,742 to 9,833 participants in the primary month-8-or-later window; these counts are lower than the measurement-availability counts in **Supplementary Table 4** because they additionally require non-missing Model 3 covariates. FPG had fewer post-stabilization observations than HbA1c or blood-pressure outcomes, which may have contributed to wider uncertainty in the subgroup trajectories; short-term biological and behavioral variation in FPG may also have contributed. Sensitivity analyses using all visits and alternative start points at months 4, 8, and 12 showed similar directions for HbA1c, FPG, and LDL; the DBP association was attenuated when analyses began at month 12 (**Supplementary Table 5**). The same-sample depression sensitivity showed modest attenuation for HbA1c, FPG, and LDL and little change for DBP (**Supplementary Table 6**). Additional adjustment for insurance coverage and drug-benefit status did not materially change the main pattern; baseline MMSE was available only in the ACCORD-MIND subset and was therefore used as a limited subset sensitivity analysis rather than a primary covariate (**Supplementary Table 7**). Descriptive RCT subgroup trajectories were broadly consistent with the main pattern (**Supplementary Figure 3**).

**Table 2:** Association between living alone and metabolic control levels during follow-up.

Outcome	Participants, n	Observations, n	Model 1 Beta (95% CI)	P-value	Model 2 Beta (95% CI)	P-value	Model 3 Beta (95% CI)	P-value
HbA1c (%)	9,797	107,519	0.06 (0.02, 0.11)	0.006	0.07 (0.03, 0.10)	<0.001	0.06 (0.03, 0.10)	0.001
FPG (mg/dL)	9,822	50,138	1.57 (-0.21, 3.34)	0.083	2.69 (1.15, 4.24)	0.001	2.28 (0.72, 3.83)	0.004
SBP (mmHg)	9,833	141,040	0.42 (-0.17, 1.02)	0.160	0.27 (-0.27, 0.81)	0.321	0.28 (-0.26, 0.83)	0.309
DBP (mmHg)	9,833	141,022	-0.01 (-0.42, 0.39)	0.944	0.48 (0.12, 0.84)	0.009	0.40 (0.05, 0.75)	0.027
LDL (mg/dL)	9,742	44,732	3.11 (1.73, 4.48)	<0.001	1.83 (0.51, 3.14)	0.006	1.77 (0.45, 3.09)	0.009
HDL (mg/dL)	9,742	44,733	2.00 (1.44, 2.56)	<0.001	0.12 (-0.39, 0.63)	0.645	0.29 (-0.22, 0.79)	0.263

Beta represents the mean difference for participants living alone vs. not living alone (reference), estimated by linear mixed-effects models with random intercepts. Model 1 included living-alone status only. Model 2 adjusted for age, sex, race, education, and domain-specific randomized treatment arm(s): glycemia arm for HbA1c and FPG, glycemia plus blood-pressure arm for SBP and DBP, and glycemia plus lipid arm for LDL and HDL. For BP and lipid outcomes, participants not enrolled in the corresponding factorial trial were retained with a not-enrolled indicator. Model 3 further adjusted for BMI, diabetes duration, CVD history, heart failure, depression, smoking, and alcohol consumption. Participant and observation counts are shown for the complete-case fully adjusted primary repeated-measures analytic window (month 8 or later). **Supplementary Table 4** reports measurement-availability counts before covariate-based complete-case deletion, with outcome-specific inclusion counts and exclusions relative to the analytic-cohort denominators of 8,171 participants not living alone and 2,078 participants living alone.

CI, confidence interval; DBP, diastolic blood pressure; FPG, fasting plasma glucose; HbA1c, glycated hemoglobin; HDL, high-density lipoprotein; LDL, low-density lipoprotein; SBP, systolic blood pressure.

### Metabolic variability

Variability findings were most consistent for blood pressure (**Table 3**). In fully adjusted models, living alone was associated with higher SBP variability (SD beta 0.60, 95% CI 0.38 to 0.82; ARV beta 0.65, 95% CI 0.40 to 0.89; both P<0.001) and higher DBP variability (SD beta 0.20, 95% CI 0.08 to 0.31; ARV beta 0.23, 95% CI 0.10 to 0.36; both P<0.001). Living alone was also associated with higher HbA1c SD (beta 0.018, 95% CI 0.003 to 0.032; P = 0.019), whereas the association with HbA1c ARV was borderline (beta 0.014, 95% CI -0.001 to 0.028; P = 0.059); therefore, HbA1c variability findings were not consistent across both variability metrics. LDL variability did not differ by living-alone status, whereas HDL SD and ARV were modestly higher among participants living alone.

**Table 3:** Association between living alone and visit-to-visit variability of metabolic parameters.

Outcome	Measure	Participants, n	Measurements, median (IQR)	Follow-up, median (IQR), months	Model 3 Beta (95% CI)	P-value
HbA1c (%)	SD	9,463	11 (9-14)	44 (36-56)	0.018 (0.003, 0.032)	0.019
	ARV	9,463	11 (9-14)	44 (36-56)	0.014 (-0.001, 0.028)	0.059
FPG (mg/dL)	SD	9,324	5 (4-6)	40 (28-52)	0.60 (-0.37, 1.56)	0.226
	ARV	9,324	5 (4-6)	40 (28-52)	0.40 (-0.79, 1.59)	0.513
SBP (mmHg)	SD	9,614	13 (10-17)	48 (36-56)	0.60 (0.38, 0.82)	<0.001
	ARV	9,614	13 (10-17)	48 (36-56)	0.65 (0.40, 0.89)	<0.001
DBP (mmHg)	SD	9,614	13 (10-17)	48 (36-56)	0.20 (0.08, 0.31)	0.001
	ARV	9,614	13 (10-17)	48 (36-56)	0.23 (0.10, 0.36)	<0.001
LDL (mg/dL)	SD	9,083	5 (4-5)	40 (36-52)	0.04 (-0.53, 0.61)	0.889
	ARV	9,083	5 (4-5)	40 (36-52)	0.10 (-0.57, 0.77)	0.769
HDL (mg/dL)	SD	9,084	5 (4-5)	40 (36-52)	0.16 (0.02, 0.29)	0.020
	ARV	9,084	5 (4-5)	40 (36-52)	0.20 (0.05, 0.35)	0.010

Beta represents the difference in variability measures for participants living alone vs. not living alone (reference), estimated by linear regression models.

Model 3: Adjusted for age, sex, race, education, treatment assignment, BMI, diabetes duration, CVD history, heart failure, depression, smoking, alcohol consumption, and the corresponding mean level of each parameter. For HbA1c and FPG, treatment assignment includes glycemia randomization (intensive vs. standard). For SBP and DBP, treatment assignment additionally includes blood pressure randomization (intensive BP, standard BP, or not enrolled in BP trial). For LDL and HDL, treatment assignment additionally includes lipid randomization (fenofibrate, placebo, or not enrolled in lipid trial).

ARV, average real variability; CI, confidence interval; DBP, diastolic blood pressure; FPG, fasting plasma glucose; HbA1c, glycated hemoglobin; HDL, high-density lipoprotein; LDL, low-density lipoprotein; SBP, systolic blood pressure; SD, standard deviation.

### Medication adherence and treatment management

Medication-management patterns are summarized in **Table 4**. Participants living alone had a slightly lower mean number of oral glucose-lowering medication classes during follow-up (2.26 vs 2.34; adjusted beta -0.04, 95% CI -0.08 to 0.00; P = 0.082). Suboptimal oral glucose-medication adherence was descriptively more common among participants living alone (12.2% vs 11.0%), but the adjusted association did not meet the conventional P<0.05 threshold (adjusted OR 1.08, 95% CI 1.00 to 1.17; P = 0.052). Medication discontinuation, new or modified oral therapy, and hypoglycemia-related adjustment did not differ meaningfully by living-alone status.

**Table 4:** Living alone and medication-management patterns during follow-up.

Medication domain	Measure	Not living alone	Living alone	Adjusted estimate (95% CI)	P-value
Glucose-lowering medication management	Oral glucose-lowering medication classes, mean (SD)	2.34 (1.00)	2.26 (1.00)	Beta -0.04 (-0.08, 0.00)	0.082
	Suboptimal oral medication adherence, n/visits (%)	25,446/231,555 (11.0%)	6,927/56,961 (12.2%)	OR 1.08 (1.00, 1.17)	0.052
	Oral medication discontinued, n/visits (%)	16,027/231,555 (6.9%)	4,132/56,961 (7.3%)	OR 1.02 (0.98, 1.07)	0.277
	Oral dose modified or new medication, n/visits (%)	18,643/231,555 (8.1%)	4,705/56,961 (8.3%)	OR 1.01 (0.97, 1.06)	0.636
	Hypoglycemia-related adjustment, n/visits (%)	2,420/231,555 (1.0%)	605/56,961 (1.1%)	OR 0.98 (0.86, 1.12)	0.772
Blood-pressure medication management	Protocol-indicated therapy change, n/visits (%)	22,003/49,362 (44.6%)	6,418/13,665 (47.0%)	OR 1.06 (0.95, 1.17)	0.296
	BP medication added, n/visits (%)	2,383/49,362 (4.8%)	723/13,665 (5.3%)	OR 1.06 (0.95, 1.19)	0.294
	BP medication changed, n/visits (%)	9,366/49,362 (19.0%)	2,739/13,665 (20.0%)	OR 1.08 (0.98, 1.19)	0.109
	No change due to participant refusal, n/visits (%)	1,905/49,362 (3.9%)	567/13,665 (4.1%)	OR 1.01 (0.85, 1.21)	0.871
	No change due to adherence problem, n/visits (%)	2,052/49,362 (4.2%)	629/13,665 (4.6%)	OR 1.07 (0.93, 1.24)	0.322
	No change due to intolerance/adverse experience, n/visits (%)	724/49,362 (1.5%)	205/13,665 (1.5%)	OR 0.98 (0.76, 1.26)	0.86
Lipid medication management	High-dose simvastatin at visit exit, n/visits (%)	16,111/62,905 (25.6%)	3,408/14,209 (24.0%)	OR 1.08 (0.94, 1.24)	0.292
	Suboptimal simvastatin adherence, n/visits (%)	3,450/57,489 (6.0%)	871/12,866 (6.8%)	OR 1.08 (0.93, 1.26)	0.3
	Suboptimal fenofibrate/placebo adherence, n/visits (%)	6,408/55,178 (11.6%)	1,585/12,365 (12.8%)	OR 1.09 (0.94, 1.26)	0.265
	No simvastatin due to refusal/intolerance/adverse experience, n/visits (%)	1,350/62,905 (2.1%)	314/14,209 (2.2%)	OR 0.96 (0.72, 1.28)	0.772
	Other lipid-lowering medication, n/visits (%)	2,991/62,905 (4.8%)	535/14,209 (3.8%)	OR 0.76 (0.62, 0.92)	0.005

Visits through 60 months were included. Adjusted estimates compare participants living alone with those not living alone.

Glucose-medication adherence used the ACCORD adherence format: 1=all/almost all (80-100%), 2=some (1-79%), 3=none, 4=>prescribed. Suboptimal adherence was defined as 2 or 3.

Action codes for oral glucose-lowering medications were interpreted as 1=no change, 2=discontinued, 3=dose modified, and 4=new medication.

BP medication indicators were interpreted using the ACCORD CRF format file: 1=yes and 2=no where applicable; yes-only reason variables were coded as no when missing.

Lipid-medication adherence used the format 1=all/almost all or >prescribed, 2=some, and 3=none; suboptimal adherence was defined as 2 or 3.

Models adjusted for age, sex, race, education, BMI, diabetes duration, randomized treatment arm(s), baseline CVD, heart failure, depression, smoking, alcohol use, and visit where applicable, with participant-clustered robust standard errors.

In blood-pressure medication analyses, participants living alone had a slightly higher proportion of visits with protocol-indicated therapy change (47.0% vs 44.6%), medication addition (5.3% vs 4.8%), and medication change (20.0% vs 19.0%), but adjusted associations were not statistically significant. In lipid medication analyses, suboptimal simvastatin adherence and suboptimal fenofibrate/placebo adherence were numerically more frequent among participants living alone but not statistically significant. Other lipid-lowering medication use was lower among participants living alone (3.8% vs 4.8%; adjusted OR 0.76, 95% CI 0.62 to 0.92; P = 0.005).

### Lagged medication-outcome associations

In lagged interval models (**Table 5**), suboptimal oral glucose-medication adherence was associated with higher subsequent HbA1c (beta 0.08%, 95% CI 0.07 to 0.10; P<0.001) and higher subsequent FPG (beta 6.19 mg/dL, 95% CI 4.70 to 7.69; P<0.001), after adjustment for the current metabolic value and covariates. Greater oral glucose-lowering medication class count was associated with slightly lower subsequent HbA1c, whereas new or modified oral glucose-lowering therapy was associated with higher subsequent HbA1c (beta 0.21%, 95% CI 0.19 to 0.22), likely reflecting treatment intensification in response to worse prior control.

**Table 5:** Lagged associations of medication-management patterns with subsequent metabolic control.

Domain	Medication exposure during prior 4-month interval	Next metabolic outcome	Modeled intervals/participants	Exposure prevalence or mean (SD)	Adjusted beta (95% CI)	P-value
Glucose-lowering medication	Suboptimal oral glucose-medication adherence	Next HbA1c, %	92,809/9,644	18,111/93,771 (19.3%)	0.08 (0.07, 0.10)	<0.001
	Suboptimal oral glucose-medication adherence	Next FPG, mg/dL	26,670/9,471	4,885/26,949 (18.1%)	6.19 (4.70, 7.69)	<0.001
	Oral glucose-lowering medication classes, mean during interval	Next HbA1c, %	97,462/9,835	2.17 (0.99)	-0.01 (-0.02, -0.01)	<0.001
	New or modified oral glucose-lowering medication	Next HbA1c, %	97,462/9,835	13,187/98,468 (13.4%)	0.21 (0.19, 0.22)	<0.001
Blood-pressure medication	Protocol-indicated BP therapy change	Next SBP, mmHg	27,117/2,531	15,722/27,421 (57.3%)	14.05 (13.71, 14.39)	<0.001
	BP medication added or changed	Next SBP, mmHg	27,117/2,531	9,136/27,421 (33.3%)	9.85 (9.45, 10.24)	<0.001
	No BP change due to refusal or adherence problem	Next SBP, mmHg	27,117/2,531	3,912/27,421 (14.3%)	8.17 (7.69, 8.64)	<0.001
	No BP change due to refusal or adherence problem	Next DBP, mmHg	27,117/2,531	3,912/27,421 (14.3%)	2.42 (2.12, 2.72)	<0.001
Lipid medication	Suboptimal simvastatin adherence	Next LDL, mg/dL	14,359/5,163	687/14,493 (4.7%)	14.97 (12.73, 17.21)	<0.001
	Low-dose or no simvastatin at interval exit	Next LDL, mg/dL	14,354/5,162	1,519/14,488 (10.5%)	11.00 (9.61, 12.40)	<0.001
	Other lipid-lowering medication use	Next LDL, mg/dL	15,059/5,315	183/15,196 (1.2%)	-1.19 (-5.63, 3.25)	0.598
	Suboptimal fenofibrate/placebo adherence	Next HDL, mg/dL	14,632/5,269	2,033/14,766 (13.8%)	-0.02 (-0.33, 0.29)	0.913

Medication-management variables were summarized during each 4-month interval and modeled against the next metabolic measurement. Modeled intervals/participants are intervals included in the lagged regression after requiring the next metabolic outcome and model covariates; exposure-prevalence denominators are intervals with nonmissing exposure data and may therefore be slightly larger.

For adherence exposures, intervals with no non-missing adherence information were coded as missing and excluded from the corresponding model.

Linear models adjusted for the current value of the same metabolic outcome, living-alone status, visit interval, age, sex, race, education, BMI, diabetes duration, baseline CVD, heart failure, depression, smoking, alcohol use, and domain-specific randomized arm(s): glycemia arm for glucose-medication models, glycemia plus BP arm for BP-medication models, and glycemia plus lipid arm for lipid-medication models.

Robust standard errors were clustered by participant. Positive beta values indicate higher subsequent values of the metabolic outcome.

These analyses are exploratory and should not be interpreted as causal mediation because treatment changes can respond to prior disease severity.

Blood-pressure treatment-management indicators were strongly associated with higher subsequent SBP, including protocol-indicated BP therapy change, medication addition or change, and no BP change due to refusal or adherence problems. These associations likely reflect underlying treatment need and residual disease severity rather than a causal effect of treatment management on higher BP. In lipid analyses, suboptimal simvastatin adherence was associated with higher subsequent LDL cholesterol (beta 14.97 mg/dL, 95% CI 12.73 to 17.21; P<0.001), as was low-dose or no simvastatin use at interval exit (beta 11.00 mg/dL, 95% CI 9.61 to 12.40; P<0.001). Other lipid-lowering medication use was not associated with subsequent LDL, and suboptimal fenofibrate/placebo adherence was not associated with subsequent HDL. Results were directionally consistent when poor adherence was defined more strictly as none of the time only (**Supplementary Table 8**).

### Exploratory microvascular and neuropathy outcomes

In exploratory Cox models (**Table 6**), living alone was not significantly associated with the UKPDS composite outcome, nephropathy outcomes, or most retinopathy outcomes. Nominal associations were observed for neuropathy defined by MNSI score >2.0 (61.8% vs 56.0%; adjusted HR 1.14, 95% CI 1.04 to 1.24; P = 0.004) and ankle jerk loss (50.9% vs 46.6%; adjusted HR 1.11, 95% CI 1.02 to 1.21; P = 0.020). These neuropathy findings did not remain below the family-specific false-discovery-rate threshold for the microvascular/neuropathy outcome family (MNSI q=0.068; ankle jerk q=0.162) and were therefore retained as exploratory signals. Exploratory supplementary analyses showed that glycemic mean and variability did not materially attenuate these neuropathy associations (**Supplementary Table 9**), whereas baseline depression partially attenuated the living-alone association (**Supplementary Table 10**). Full false-discovery-rate sensitivity results are shown in **Supplementary Table 11**.

**Table 6:** Association of living alone with microvascular outcomes.

Domain	Outcome	Not living alone events/N (%)	Living alone events/N (%)	Adjusted HR (95% CI)	P-value
Composite	UKPDS composite: nephropathy outcome 3 or retinopathy outcome 1	923/8,155 (11.3%)	219/2,077 (10.5%)	0.94 (0.81, 1.10)	0.439
	UKPDS + neuropathy composite	3,177/8,155 (39.0%)	825/2,077 (39.7%)	1.04 (0.96, 1.13)	0.325
Nephropathy	SCr doubling or >20 mL/min decrease in eGFR	4,729/8,032 (58.9%)	1,169/2,042 (57.2%)	1.02 (0.95, 1.09)	0.564
	Development of macroalbuminuria (UAlb >=300)	491/7,080 (6.9%)	135/1,740 (7.8%)	1.13 (0.93, 1.38)	0.209
	Renal failure, ESRD/dialysis, or SCr >3.3	236/8,155 (2.9%)	53/2,077 (2.6%)	0.95 (0.70, 1.29)	0.75
	Any nephropathy outcome 1-3	4,958/8,155 (60.8%)	1,225/2,077 (59.0%)	1.02 (0.95, 1.08)	0.625
	Development of microalbuminuria (UAlb >=30)	1,222/5,257 (23.2%)	327/1,265 (25.8%)	1.09 (0.96, 1.24)	0.189
Retinopathy	Photocoagulation or vitrectomy	723/7,872 (9.2%)	173/1,975 (8.8%)	0.93 (0.78, 1.10)	0.399
	Cataract extraction	1,155/7,872 (14.7%)	316/1,975 (16.0%)	0.94 (0.83, 1.07)	0.37
	3-line change in visual acuity	2,814/7,624 (36.9%)	736/1,897 (38.8%)	1.04 (0.96, 1.13)	0.315
	3-line change in visual acuity using VAS	2,308/7,624 (30.3%)	619/1,897 (32.6%)	1.07 (0.97, 1.17)	0.165
	Severe vision loss (SF <20/200)	604/7,624 (7.9%)	168/1,897 (8.9%)	1.04 (0.87, 1.24)	0.662
Neuropathy	MNSI score >2.0	2,541/4,538 (56.0%)	672/1,088 (61.8%)	1.14 (1.04, 1.24)	0.004
	Vibratory sensation loss	1,618/6,744 (24.0%)	413/1,699 (24.3%)	1.04 (0.93, 1.16)	0.496
	Ankle jerk loss	2,459/5,273 (46.6%)	667/1,310 (50.9%)	1.11 (1.02, 1.21)	0.02
	Pressure sensation loss	953/7,326 (13.0%)	246/1,842 (13.4%)	1.07 (0.93, 1.23)	0.368

Hazard ratios compare participants living alone with those not living alone.

Cox models adjusted for age, sex, race, education, BMI, diabetes duration, randomized treatment arm(s), baseline CVD, heart failure, depression, smoking, and alcohol use.

Microvascular outcome definitions are from the ACCORD microvascular outcome dataset.

## Discussion

In this secondary analysis of ACCORD, living alone was associated with small differences in long-term glycemic and lipid levels and with greater variability in several cardiometabolic indicators. The most consistent variability signals were observed for blood pressure, while mean-level differences were most apparent for HbA1c, FPG, DBP, and LDL in nominal analyses. The wider FPG dispersion and uncertainty should not be attributed only to measurement density; it may also reflect fasting status, short-term medication changes, and day-to-day glucose fluctuation. Denser glucose assessment, including continuous glucose-monitoring approaches, could help clarify this pattern in future studies (15). Living alone was also linked to selected medication-management patterns, particularly a borderline higher frequency of suboptimal oral glucose-medication adherence and lower use of other lipid-lowering medications.

The mean-level estimates were statistically detectable but small. Differences of approximately 0.06% in HbA1c, 2 mg/dL in FPG, less than 1 mmHg in DBP, and about 2 mg/dL in LDL should not be interpreted as large individual-level differences in metabolic control. Their relevance is mainly contextual: in ACCORD, frequent visits and protocolized risk-factor management likely compressed between-group differences, and the more consistent signal was variability rather than large shifts in average levels. These findings therefore identify living arrangement as a potential marker of day-to-day management instability rather than evidence that living alone produces clinically large metabolic deterioration.

The medication analyses provide a behavioral pathway for interpreting the metabolic findings. Suboptimal oral glucose-medication adherence was followed by higher HbA1c and FPG, and suboptimal simvastatin adherence was followed by markedly higher LDL. Because the association between living alone and suboptimal oral glucose-medication adherence was borderline, these results should be read as supportive context rather than proof of mediation. The stricter poor-adherence definition gave similar results for glucose and simvastatin adherence. By contrast, the blood-pressure medication results are better read as markers of treatment need and clinical response: patients with uncontrolled BP were more likely to have protocol-indicated therapy changes or medication intensification, which explains their higher subsequent BP.

The exploratory neuropathy findings were notable but should be interpreted cautiously. Living alone showed nominal associations with MNSI-defined neuropathy and ankle jerk loss, but these associations did not remain below the family-specific false-discovery-rate threshold and were not accompanied by broad nephropathy or retinopathy associations. Additional analyses showed little attenuation after adjustment for HbA1c/FPG mean and variability, whereas adjustment for depression weakened the association. This pattern argues against a purely glycemic pathway for neuropathy. Depression was substantially more common at baseline among participants living alone and could influence neuropathy through symptom perception, activity limitation, and care engagement (16–18). The mechanism remains uncertain and requires further study.

For clinical practice, living arrangement is not a substitute for formal assessment of social support, loneliness, depression, cognition, or medication adherence, but it is available at the point of care (19). Asking whether a patient lives alone can help clinicians decide when to check medication routines more closely, review home blood-pressure patterns, or look for visit-to-visit instability in glycemia or lipids.

### Strengths and limitations

Strengths include the large trial population, repeated measurements over time, standardized follow-up, detailed medication-management records, and simultaneous evaluation of glycemic, blood-pressure, lipid, medication, and microvascular domains. Several limitations should be considered. First, living-alone status was measured only at baseline and did not capture marriage, divorce, widowhood, moving, institutionalization, or other changes in household structure during follow-up, creating possible exposure misclassification. Second, the ACCORD household variable identified whether participants lived with another adult but did not separately identify those living with children or dependents only. Third, living alone is not equivalent to loneliness, social isolation, or lack of social support, and the audited ACCORD public-use data did not provide detailed full-cohort measures of marital status, income, employment, loneliness, social support, or household changes for complete social-context confounding control. Insurance coverage, drug-benefit category, and baseline MMSE were examined in sensitivity analyses when available, but MMSE was limited to the ACCORD-MIND subset and could not serve as a full-cohort cognitive adjustment. Although medication records were analyzed separately, an aggregate baseline medication-burden construct was not incorporated into the primary confounding set. Depression may also lie partly on the causal pathway linking household context and self-management. Fourth, the analysis is observational with respect to living alone, so residual confounding is possible. Fifth, medication adherence variables were derived from visit-recorded participant estimates rather than pharmacy refill data, pill counts, or electronic monitoring, and do not capture all real-world adherence behavior. Sixth, the ACCORD population consisted of patients with type 2 diabetes at high cardiovascular risk receiving protocolized trial care, which limits generalizability. Finally, multiple metabolic, medication, and microvascular comparisons were conducted; findings not robust to false-discovery-rate sensitivity analyses, especially the neuropathy outcomes, should be considered exploratory.

## Conclusion

Among adults with type 2 diabetes enrolled in ACCORD, living alone was associated with small differences in glycemic and lipid levels, greater variability in selected cardiometabolic indicators, and selected medication-management patterns linked to subsequent metabolic control. Living arrangement is simple to ascertain, and in this trial it identified patients with a pattern of long-term self-management instability even under close protocolized follow-up. These data support paying closer attention to medication routines, social context, and depression among patients with diabetes who live alone. Living alone is not a direct measure of loneliness or social support, and the exploratory neuropathy signals require confirmation.

## Data Availability

The ACCORD data analyzed in this study are available through the National Heart, Lung, and Blood Institute BioLINCC repository, subject to application, approval, and applicable data-use conditions.
