# Supplementary material for: Living alone, long-term cardiometabolic stability, and medication management in adults with type 2 diabetes: a secondary analysis of the ACCORD trial
Source: Front Endocrinol (Lausanne). 2026 Jul 31;17:1906084. doi: 10.3389/fendo.2026.1906084 (PMC13472925; doi:10.3389/fendo.2026.1906084)
Supplement: Supplementary file 1 [file SupplementaryFile1.docx]

Supplementary Material

Living Alone, Long-term Cardiometabolic Stability, and Medication Management in Adults With Type 2 Diabetes: A Secondary Analysis of the ACCORD Trial

Contents

| **Item** | **Title** |
| --- | --- |
| Supplementary Table 1 | Definitions of exposure, medication-record variables, and microvascular outcomes |
| Supplementary Table 2 | Available baseline living-status categories |
| Supplementary Table 3 | Baseline distributions of additional measured covariates |
| Supplementary Table 4 | Outcome-specific measurement availability and exclusions by living-alone status |
| Supplementary Table 5 | Fully adjusted longitudinal model sensitivity by analytic start point |
| Supplementary Table 6 | Same-sample depression sensitivity for the main longitudinal outcomes |
| Supplementary Table 7 | Sensitivity adjustment for insurance, drug benefits, and MMSE |
| Supplementary Table 8 | Sensitivity analysis using strict poor-adherence definitions |
| Supplementary Table 9 | Exploratory glycemic mean and variability attenuation analysis for neuropathy outcomes |
| Supplementary Table 10 | Exploratory depression attenuation analysis for neuropathy outcomes |
| Supplementary Table 11 | Benjamini-Hochberg false-discovery-rate sensitivity using raw model P values |
| Supplementary Figure 1 | Conceptual framework |
| Supplementary Figure 2 | Forest plot of fully adjusted model estimates |
| Supplementary Figure 3 | RCT subgroup longitudinal trajectories |

# Supplementary Tables

## Supplementary Table 1. Definitions of exposure, medication-record variables, and microvascular outcomes

| **Domain** | **Variable or measure** | **Definition used in this analysis** |
| --- | --- | --- |
| Exposure | Living-alone status | Baseline living arrangement. Participants were classified as living alone when the ACCORD baseline living-arrangement variable indicated living without another adult; participants not living alone were the reference group. |
| Longitudinal metabolic outcomes | HbA1c, FPG, SBP, DBP, LDL cholesterol, HDL cholesterol | Repeated protocol and follow-up measurements from ACCORD domain-specific analysis datasets. HbA1c was expressed as %, and FPG, LDL, and HDL were expressed as mg/dL; SBP and DBP were expressed as mmHg. |
| Metabolic variability | Standard deviation (SD) | Within-person SD of repeated values for each metabolic indicator. Participants were required to have at least three non-missing measurements for each indicator. |
| Metabolic variability | Average real variability (ARV) | Mean absolute difference between consecutive non-missing visits for each metabolic indicator. |
| Medication records | Glucose-medication records | Longitudinal glycemia medication logs derived from ACCORD case-report forms. These records captured prescribed glucose-lowering medications, medication class information, action or modification codes, reason codes, and participant-estimated adherence at protocol visits. |
| Medication records | Blood-pressure medication records | Blood-pressure trial medication-management records derived from ACCORD case-report forms. Analyses used protocol-indicated therapy change, medication addition or change, and no-change reason codes, including participant refusal, adherence problems, intolerance, or adverse experience. |
| Medication records | Lipid medication records | Lipid trial medication-management records derived from ACCORD case-report forms. Analyses used simvastatin and fenofibrate/placebo adherence, simvastatin dose or nonuse indicators, intolerance or refusal indicators, and other lipid-lowering medication use. |
| Medication adherence | Oral glucose-medication adherence | Participant-estimated adherence to prescribed oral glucose-lowering therapy. ACCORD codes were: 1, all or almost all of the time (80-100%); 2, some of the time (1-79%); 3, none of the time (0%); 4, more than prescribed (>100%). Suboptimal adherence was defined as code 2 or 3. |
| Medication adherence | Simvastatin and fenofibrate/placebo adherence | Participant-estimated adherence to lipid trial therapy. ACCORD lipid adherence codes were: 1, all/almost all use or more than prescribed; 2, some of the time; 3, none of the time. Suboptimal adherence was defined as code 2 or 3. |
| Medication adherence sensitivity | Strict poor adherence | Sensitivity definition using none of the time only: code 3 for oral glucose-medication adherence, simvastatin adherence, or fenofibrate/placebo adherence. |
| Microvascular composite outcomes | UKPDS composite | ACCORD microvascular outcome composite defined as nephropathy outcome 3 or retinopathy outcome 1: renal failure, ESRD/dialysis, or serum creatinine >3.3 mg/dL; or retinal photocoagulation/vitrectomy. |
| Microvascular composite outcomes | UKPDS + neuropathy composite | Expanded composite defined as the UKPDS composite plus neuropathy defined by Michigan Neuropathy Screening Instrument (MNSI) score >2.0. |
| Nephropathy outcomes | Nephropathy outcome 1 | Doubling of serum creatinine or >20 mL/min decrease in estimated glomerular filtration rate. |
| Nephropathy outcomes | Nephropathy outcome 2 | Development of macroalbuminuria, defined as urinary albumin-to-creatinine ratio >=300 mg/g. |
| Nephropathy outcomes | Nephropathy outcome 3 | Renal failure, ESRD/dialysis, or serum creatinine >3.3 mg/dL. |
| Nephropathy outcomes | Nephropathy outcome 4 | Any of nephropathy outcomes 1 through 3. |
| Nephropathy outcomes | Nephropathy outcome 5 | Development of microalbuminuria, defined as urinary albumin-to-creatinine ratio >=30 mg/g. |
| Retinopathy outcomes | Retinopathy outcome 1 | Retinal photocoagulation or vitrectomy. |
| Retinopathy outcomes | Retinopathy outcome 2 | Cataract extraction. |
| Retinopathy outcomes | Retinopathy outcome 3 | Three-line change in visual acuity, including the available VAS-based version. |
| Retinopathy outcomes | Retinopathy outcome 4 | Severe vision loss, defined as visual acuity <20/200. |
| Neuropathy outcomes | Neuropathy outcome 1 | Michigan Neuropathy Screening Instrument (MNSI) score >2.0. |
| Neuropathy outcomes | Neuropathy outcome 2 | Vibratory sensation loss. |
| Neuropathy outcomes | Neuropathy outcome 3 | Ankle jerk loss. |
| Neuropathy outcomes | Neuropathy outcome 4 | Pressure sensation loss. |

Note. Microvascular outcome definitions follow the ACCORD microvascular outcomes analysis dataset and the published ACCORD microvascular outcome framework. Medication records refer to visit-based ACCORD medication case-report-form records rather than pharmacy refill claims or electronic monitoring.

## Supplementary Table 2. Available baseline living-status categories

| **Baseline living-status category** | **n** | **Percent of randomized cohort** |
| --- | --- | --- |
| Not living alone | 8,171 | 79.7% |
| Living alone | 2,078 | 20.3% |
| Missing baseline living-status information in original randomized cohort | 2 | <0.1% |

Note. Not living alone denotes living with one or more adults at baseline; living alone denotes not living with another adult at baseline. Two randomized participants were excluded because this information was missing.

## Supplementary Table 3. Baseline distributions of additional measured covariates

| **Covariate** | **Category or statistic** | **Overall** | **Not living alone** | **Living alone** |
| --- | --- | --- | --- | --- |
| Any insurance coverage | No / uninsured or do not know | 1,461 (14.3%) | 1,151 (14.1%) | 310 (14.9%) |
| Any insurance coverage | Yes | 8,785 (85.7%) | 7,017 (85.9%) | 1,768 (85.1%) |
| Any insurance coverage | Missing | 3 (<0.1%) | 3 (<0.1%) | 0 (0.0%) |
| Prescription drug-benefit status | Yes | 7,389 (72.1%) | 5,965 (73.0%) | 1,424 (68.5%) |
| Prescription drug-benefit status | No | 1,337 (13.0%) | 993 (12.2%) | 344 (16.6%) |
| Prescription drug-benefit status | Don't know | 240 (2.3%) | 193 (2.4%) | 47 (2.3%) |
| Prescription drug-benefit status | Uninsured | 1,280 (12.5%) | 1,017 (12.4%) | 263 (12.7%) |
| Prescription drug-benefit status | Missing | 3 (<0.1%) | 3 (<0.1%) | 0 (0.0%) |
| TOTAL MMSE score | Mean among nonmissing | 27.4 (N=2,977) | 27.4 (N=2,320) | 27.4 (N=657) |

Note. Categorical rows are shown as n (%). The MMSE row is shown as mean (N) among participants in the ACCORD-MIND subset. Prescription drug-benefit status is shown using the original ACCORD CRF four-category coding: Yes, No, Don't know, and Uninsured; this source item was not recoded to match the separately derived any-insurance variable.

## Supplementary Table 4. Outcome-specific measurement availability and exclusions by living-alone status

| **Window** | **Outcome** | **Living status group** | **Participants included, n** | **Observations, n** | **Excluded participants, n (%)** |
| --- | --- | --- | --- | --- | --- |
| Month 8 onward (primary) | HbA1c | Overall | 9,903 | 108,580 | 346 (of 10,249; 3.4%) |
| Month 8 onward (primary) | HbA1c | Not living alone | 7,904 | 86,964 | 267 (of 8,171; 3.3%) |
| Month 8 onward (primary) | HbA1c | Living alone | 1,999 | 21,616 | 79 (of 2,078; 3.8%) |
| Month 8 onward (primary) | FPG | Overall | 9,928 | 50,626 | 321 (of 10,249; 3.1%) |
| Month 8 onward (primary) | FPG | Not living alone | 7,924 | 40,508 | 247 (of 8,171; 3.0%) |
| Month 8 onward (primary) | FPG | Living alone | 2,004 | 10,118 | 74 (of 2,078; 3.6%) |
| Month 8 onward (primary) | SBP | Overall | 9,941 | 142,481 | 308 (of 10,249; 3.0%) |
| Month 8 onward (primary) | SBP | Not living alone | 7,930 | 113,691 | 241 (of 8,171; 2.9%) |
| Month 8 onward (primary) | SBP | Living alone | 2,011 | 28,790 | 67 (of 2,078; 3.2%) |
| Month 8 onward (primary) | DBP | Overall | 9,941 | 142,462 | 308 (of 10,249; 3.0%) |
| Month 8 onward (primary) | DBP | Not living alone | 7,930 | 113,679 | 241 (of 8,171; 2.9%) |
| Month 8 onward (primary) | DBP | Living alone | 2,011 | 28,783 | 67 (of 2,078; 3.2%) |
| Month 8 onward (primary) | LDL | Overall | 9,846 | 45,165 | 403 (of 10,249; 3.9%) |
| Month 8 onward (primary) | LDL | Not living alone | 7,862 | 36,260 | 309 (of 8,171; 3.8%) |
| Month 8 onward (primary) | LDL | Living alone | 1,984 | 8,905 | 94 (of 2,078; 4.5%) |
| Month 8 onward (primary) | HDL | Overall | 9,846 | 45,166 | 403 (of 10,249; 3.9%) |
| Month 8 onward (primary) | HDL | Not living alone | 7,862 | 36,261 | 309 (of 8,171; 3.8%) |
| Month 8 onward (primary) | HDL | Living alone | 1,984 | 8,905 | 94 (of 2,078; 4.5%) |
| Month 12 onward (sensitivity) | HbA1c | Overall | 9,786 | 99,425 | 463 (of 10,249; 4.5%) |
| Month 12 onward (sensitivity) | HbA1c | Not living alone | 7,821 | 79,652 | 350 (of 8,171; 4.3%) |
| Month 12 onward (sensitivity) | HbA1c | Living alone | 1,965 | 19,773 | 113 (of 2,078; 5.4%) |
| Month 12 onward (sensitivity) | FPG | Overall | 9,806 | 41,010 | 443 (of 10,249; 4.3%) |
| Month 12 onward (sensitivity) | FPG | Not living alone | 7,834 | 32,847 | 337 (of 8,171; 4.1%) |
| Month 12 onward (sensitivity) | FPG | Living alone | 1,972 | 8,163 | 106 (of 2,078; 5.1%) |
| Month 12 onward (sensitivity) | SBP | Overall | 9,835 | 131,616 | 414 (of 10,249; 4.0%) |
| Month 12 onward (sensitivity) | SBP | Not living alone | 7,856 | 105,056 | 315 (of 8,171; 3.9%) |
| Month 12 onward (sensitivity) | SBP | Living alone | 1,979 | 26,560 | 99 (of 2,078; 4.8%) |
| Month 12 onward (sensitivity) | DBP | Overall | 9,835 | 131,598 | 414 (of 10,249; 4.0%) |
| Month 12 onward (sensitivity) | DBP | Not living alone | 7,856 | 105,045 | 315 (of 8,171; 3.9%) |
| Month 12 onward (sensitivity) | DBP | Living alone | 1,979 | 26,553 | 99 (of 2,078; 4.8%) |
| Month 12 onward (sensitivity) | LDL | Overall | 9,775 | 39,992 | 474 (of 10,249; 4.6%) |
| Month 12 onward (sensitivity) | LDL | Not living alone | 7,808 | 32,060 | 363 (of 8,171; 4.4%) |
| Month 12 onward (sensitivity) | LDL | Living alone | 1,967 | 7,932 | 111 (of 2,078; 5.3%) |
| Month 12 onward (sensitivity) | HDL | Overall | 9,775 | 39,993 | 474 (of 10,249; 4.6%) |
| Month 12 onward (sensitivity) | HDL | Not living alone | 7,808 | 32,061 | 363 (of 8,171; 4.4%) |
| Month 12 onward (sensitivity) | HDL | Living alone | 1,967 | 7,932 | 111 (of 2,078; 5.3%) |

Note. Excluded counts use the analytic cohort denominators of 8,171 participants not living alone and 2,078 participants living alone. The primary analysis used the month-8-or-later window.

## Supplementary Table 5. Fully adjusted longitudinal model sensitivity by analytic start point

| **Analysis window** | **Outcome** | **Beta (95% CI)** | **P value** | **Participants, n** | **Observations, n** |
| --- | --- | --- | --- | --- | --- |
| All visits | HbA1c | 0.065 (0.031, 0.099) | <0.001 | 10,243 | 128,215 |
| All visits | FPG | 2.80 (1.35, 4.25) | <0.001 | 10,245 | 70,588 |
| All visits | SBP | 0.19 (-0.34, 0.72) | 0.474 | 10,247 | 173,595 |
| All visits | DBP | 0.41 (0.07, 0.75) | 0.020 | 10,247 | 173,574 |
| All visits | LDL | 1.69 (0.47, 2.91) | 0.007 | 10,242 | 60,627 |
| All visits | HDL | 0.34 (-0.14, 0.81) | 0.166 | 10,242 | 60,629 |
| Month 4 onward | HbA1c | 0.059 (0.024, 0.093) | 0.001 | 10,043 | 117,988 |
| Month 4 onward | FPG | 2.41 (0.92, 3.91) | 0.002 | 10,060 | 60,392 |
| Month 4 onward | SBP | 0.35 (-0.19, 0.89) | 0.203 | 10,060 | 153,991 |
| Month 4 onward | DBP | 0.47 (0.12, 0.81) | 0.008 | 10,060 | 153,970 |
| Month 4 onward | LDL | 1.74 (0.43, 3.04) | 0.009 | 9,915 | 50,433 |
| Month 4 onward | HDL | 0.27 (-0.23, 0.76) | 0.289 | 9,915 | 50,434 |
| Month 8 onward (primary) | HbA1c | 0.062 (0.026, 0.098) | 0.001 | 9,903 | 108,580 |
| Month 8 onward (primary) | FPG | 2.28 (0.72, 3.83) | 0.004 | 9,928 | 50,626 |
| Month 8 onward (primary) | SBP | 0.28 (-0.26, 0.83) | 0.309 | 9,941 | 142,481 |
| Month 8 onward (primary) | DBP | 0.40 (0.05, 0.75) | 0.027 | 9,941 | 142,462 |
| Month 8 onward (primary) | LDL | 1.77 (0.45, 3.09) | 0.009 | 9,846 | 45,165 |
| Month 8 onward (primary) | HDL | 0.29 (-0.22, 0.79) | 0.263 | 9,846 | 45,166 |
| Month 12 onward (sensitivity) | HbA1c | 0.063 (0.026, 0.100) | 0.001 | 9,786 | 99,425 |
| Month 12 onward (sensitivity) | FPG | 2.26 (0.64, 3.89) | 0.006 | 9,806 | 41,010 |
| Month 12 onward (sensitivity) | SBP | 0.25 (-0.30, 0.80) | 0.373 | 9,835 | 131,616 |
| Month 12 onward (sensitivity) | DBP | 0.35 (-0.01, 0.70) | 0.057 | 9,835 | 131,598 |
| Month 12 onward (sensitivity) | LDL | 1.92 (0.58, 3.26) | 0.005 | 9,775 | 39,992 |
| Month 12 onward (sensitivity) | HDL | 0.24 (-0.27, 0.75) | 0.362 | 9,775 | 39,993 |

Note. Models used the fully adjusted covariate set and domain-specific randomized treatment arm indicators.

## Supplementary Table 6. Same-sample depression sensitivity for the main longitudinal outcomes

| **Outcome** | **Model** | **Beta (95% CI)** | **P value** | **Participants, n** | **Observations, n** |
| --- | --- | --- | --- | --- | --- |
| HbA1c | Without baseline depression | 0.066 (0.030, 0.102) | <0.001 | 9,797 | 107,519 |
| HbA1c | With baseline depression | 0.062 (0.026, 0.098) | 0.001 | 9,797 | 107,519 |
| FPG | Without baseline depression | 2.53 (0.98, 4.08) | 0.001 | 9,822 | 50,138 |
| FPG | With baseline depression | 2.28 (0.72, 3.83) | 0.004 | 9,822 | 50,138 |
| SBP | Without baseline depression | 0.25 (-0.29, 0.80) | 0.361 | 9,833 | 141,040 |
| SBP | With baseline depression | 0.28 (-0.26, 0.83) | 0.309 | 9,833 | 141,040 |
| DBP | Without baseline depression | 0.40 (0.04, 0.75) | 0.027 | 9,833 | 141,022 |
| DBP | With baseline depression | 0.40 (0.05, 0.75) | 0.027 | 9,833 | 141,022 |
| LDL | Without baseline depression | 1.83 (0.51, 3.15) | 0.007 | 9,742 | 44,732 |
| LDL | With baseline depression | 1.77 (0.45, 3.09) | 0.009 | 9,742 | 44,732 |
| HDL | Without baseline depression | 0.26 (-0.25, 0.76) | 0.318 | 9,742 | 44,733 |
| HDL | With baseline depression | 0.29 (-0.22, 0.79) | 0.263 | 9,742 | 44,733 |

Note. The without-depression model used the fully adjusted covariate set except baseline depression; the with-depression model added baseline depression as the only incremental covariate.

## Supplementary Table 7. Sensitivity adjustment for insurance, drug benefits, and MMSE

| **Outcome** | **Sensitivity model** | **Beta (95% CI)** | **P value** | **Participants, n** | **Observations, n** |
| --- | --- | --- | --- | --- | --- |
| HbA1c | Base model in same sample as insurance/drug-benefit model | 0.062 (0.026, 0.098) | 0.001 | 9,795 | 107,495 |
| HbA1c | Additionally adjusted for insurance and drug benefit | 0.064 (0.028, 0.100) | 0.001 | 9,795 | 107,495 |
| HbA1c | Base model in ACCORD-MIND subset | 0.034 (-0.030, 0.099) | 0.296 | 2,889 | 28,189 |
| HbA1c | Additionally adjusted for baseline MMSE in ACCORD-MIND subset | 0.036 (-0.029, 0.100) | 0.277 | 2,889 | 28,189 |
| FPG | Base model in same sample as insurance/drug-benefit model | 2.28 (0.73, 3.83) | 0.004 | 9,820 | 50,126 |
| FPG | Additionally adjusted for insurance and drug benefit | 2.25 (0.69, 3.80) | 0.005 | 9,820 | 50,126 |
| FPG | Base model in ACCORD-MIND subset | 2.73 (0.03, 5.44) | 0.048 | 2,897 | 13,265 |
| FPG | Additionally adjusted for baseline MMSE in ACCORD-MIND subset | 2.77 (0.06, 5.48) | 0.045 | 2,897 | 13,265 |
| SBP | Base model in same sample as insurance/drug-benefit model | 0.28 (-0.26, 0.83) | 0.309 | 9,831 | 141,014 |
| SBP | Additionally adjusted for insurance and drug benefit | 0.23 (-0.31, 0.78) | 0.397 | 9,831 | 141,014 |
| DBP | Base model in same sample as insurance/drug-benefit model | 0.40 (0.05, 0.75) | 0.027 | 9,831 | 140,996 |
| DBP | Additionally adjusted for insurance and drug benefit | 0.37 (0.02, 0.72) | 0.038 | 9,831 | 140,996 |
| LDL | Base model in same sample as insurance/drug-benefit model | 1.77 (0.44, 3.09) | 0.009 | 9,740 | 44,720 |
| LDL | Additionally adjusted for insurance and drug benefit | 1.67 (0.35, 2.99) | 0.013 | 9,740 | 44,720 |
| HDL | Base model in same sample as insurance/drug-benefit model | 0.29 (-0.22, 0.79) | 0.267 | 9,740 | 44,721 |
| HDL | Additionally adjusted for insurance and drug benefit | 0.31 (-0.19, 0.82) | 0.225 | 9,740 | 44,721 |

Note. MMSE was available only for the ACCORD-MIND baseline subset, so those models are subset sensitivity analyses.

## Supplementary Table 8. Sensitivity analysis using strict poor-adherence definitions

| **Domain** | **Medication exposure during prior 4-month interval** | **Next metabolic outcome** | **Modeled intervals / participants** | **Exposure prevalence or mean (SD)** | **Adjusted beta (95% CI)** | **P-value** |
| --- | --- | --- | --- | --- | --- | --- |
| Glucose-lowering medication | Poor oral glucose-medication adherence (none of the time only) | Next HbA1c, % | 92,809 / 9,644 | 4,720/93,771 (5.0%) | 0.11 (0.08, 0.13) | <0.001 |
| Glucose-lowering medication | Poor oral glucose-medication adherence (none of the time only) | Next FPG, mg/dL | 26,670 / 9,471 | 1,273/26,949 (4.7%) | 9.59 (6.71, 12.47) | <0.001 |
| Lipid medication | Poor simvastatin adherence (none of the time only) | Next LDL, mg/dL | 14,359 / 5,163 | 363/14,493 (2.5%) | 17.85 (14.65, 21.04) | <0.001 |
| Lipid medication | Poor fenofibrate/placebo adherence (none of the time only) | Next HDL, mg/dL | 14,632 / 5,269 | 1,742/14,766 (11.8%) | 0.06 (-0.27, 0.39) | 0.722 |

Note. Strict poor adherence was defined as reporting medication use/adherence "none of the time": comp=3 for oral glucose-lowering medications and zadhere/fadhere=3 for lipid medications.

Intervals with no non-missing adherence information were coded as missing and excluded from the corresponding model.

Linear models used the fully adjusted lagged medication-outcome covariate set and clustered robust standard errors by participant.

Modeled intervals/participants are intervals included in the lagged regression after requiring the next metabolic outcome and model covariates; exposure-prevalence denominators are intervals with nonmissing exposure data and may therefore be slightly larger.

## Supplementary Table 9. Exploratory glycemic mean and variability attenuation analysis for neuropathy outcomes

| **Outcome** | **Model** | **Participants/events** | **Living-alone HR (95% CI; P value)** | **Added glycemic metric HR (95% CI)** | **Attenuation in living-alone log-HR** |
| --- | --- | --- | --- | --- | --- |
| MNSI score >2.0 | Base | 3,321/1,206 | 1.12 (0.97, 1.29); P=0.130 | None |  |
| MNSI score >2.0 | Single metric | 3,321/1,206 | 1.12 (0.97, 1.30); P=0.125 | Mean HbA1c (per 1%): 1.11 (1.01, 1.21) | -1.3% |
| MNSI score >2.0 | Single metric | 3,321/1,206 | 1.12 (0.97, 1.29); P=0.133 | HbA1c SD (per 1%): 1.09 (0.90, 1.31) | 0.8% |
| MNSI score >2.0 | Single metric | 3,321/1,206 | 1.12 (0.97, 1.30); P=0.124 | HbA1c ARV (per 1%): 1.16 (0.99, 1.35) | -1.6% |
| MNSI score >2.0 | Single metric | 3,321/1,206 | 1.12 (0.97, 1.29); P=0.134 | Mean FPG (per 10 mg/dL): 0.99 (0.97, 1.01) | 1.2% |
| MNSI score >2.0 | Single metric | 3,321/1,206 | 1.12 (0.97, 1.29); P=0.130 | FPG SD (per 10 mg/dL): 1.00 (0.97, 1.02) | 0.1% |
| MNSI score >2.0 | Single metric | 3,321/1,206 | 1.12 (0.97, 1.29); P=0.128 | FPG ARV (per 10 mg/dL): 1.00 (0.98, 1.02) | -0.5% |
| MNSI score >2.0 | All metrics | 3,321/1,206 | 1.13 (0.97, 1.30); P=0.109 | Mean HbA1c (per 1%): 1.16 (1.04, 1.30) | -6.0% |
| MNSI score >2.0 | All metrics | 3,321/1,206 | 1.13 (0.97, 1.30); P=0.109 | HbA1c SD (per 1%): 0.77 (0.54, 1.11) | -6.0% |
| MNSI score >2.0 | All metrics | 3,321/1,206 | 1.13 (0.97, 1.30); P=0.109 | HbA1c ARV (per 1%): 1.30 (0.97, 1.74) | -6.0% |
| MNSI score >2.0 | All metrics | 3,321/1,206 | 1.13 (0.97, 1.30); P=0.109 | Mean FPG (per 10 mg/dL): 0.98 (0.95, 1.00) | -6.0% |
| MNSI score >2.0 | All metrics | 3,321/1,206 | 1.13 (0.97, 1.30); P=0.109 | FPG SD (per 10 mg/dL): 1.01 (0.96, 1.06) | -6.0% |
| MNSI score >2.0 | All metrics | 3,321/1,206 | 1.13 (0.97, 1.30); P=0.109 | FPG ARV (per 10 mg/dL): 0.99 (0.96, 1.02) | -6.0% |
| Ankle jerk loss | Base | 4,177/1,170 | 1.09 (0.94, 1.26); P=0.239 | None |  |
| Ankle jerk loss | Single metric | 4,177/1,170 | 1.09 (0.94, 1.26); P=0.240 | Mean HbA1c (per 1%): 1.00 (0.92, 1.10) | 0.1% |
| Ankle jerk loss | Single metric | 4,177/1,170 | 1.09 (0.94, 1.27); P=0.237 | HbA1c SD (per 1%): 0.96 (0.79, 1.17) | -0.6% |
| Ankle jerk loss | Single metric | 4,177/1,170 | 1.09 (0.94, 1.26); P=0.240 | HbA1c ARV (per 1%): 0.98 (0.83, 1.16) | 0.1% |
| Ankle jerk loss | Single metric | 4,177/1,170 | 1.09 (0.94, 1.27); P=0.229 | Mean FPG (per 10 mg/dL): 0.98 (0.97, 1.00) | -2.1% |
| Ankle jerk loss | Single metric | 4,177/1,170 | 1.09 (0.94, 1.27); P=0.230 | FPG SD (per 10 mg/dL): 0.99 (0.96, 1.02) | -2.0% |
| Ankle jerk loss | Single metric | 4,177/1,170 | 1.09 (0.94, 1.27); P=0.229 | FPG ARV (per 10 mg/dL): 0.99 (0.97, 1.01) | -2.3% |
| Ankle jerk loss | All metrics | 4,177/1,170 | 1.09 (0.94, 1.27); P=0.232 | Mean HbA1c (per 1%): 1.06 (0.95, 1.18) | -1.7% |
| Ankle jerk loss | All metrics | 4,177/1,170 | 1.09 (0.94, 1.27); P=0.232 | HbA1c SD (per 1%): 0.93 (0.64, 1.33) | -1.7% |
| Ankle jerk loss | All metrics | 4,177/1,170 | 1.09 (0.94, 1.27); P=0.232 | HbA1c ARV (per 1%): 1.03 (0.77, 1.40) | -1.7% |
| Ankle jerk loss | All metrics | 4,177/1,170 | 1.09 (0.94, 1.27); P=0.232 | Mean FPG (per 10 mg/dL): 0.98 (0.96, 1.00) | -1.7% |
| Ankle jerk loss | All metrics | 4,177/1,170 | 1.09 (0.94, 1.27); P=0.232 | FPG SD (per 10 mg/dL): 1.01 (0.96, 1.06) | -1.7% |
| Ankle jerk loss | All metrics | 4,177/1,170 | 1.09 (0.94, 1.27); P=0.232 | FPG ARV (per 10 mg/dL): 0.99 (0.96, 1.03) | -1.7% |

Note. This was an exploratory attenuation analysis using a 24-month landmark design.

Participants with neuropathy events or censoring before 24 months were excluded. Mean, SD, and average real variability (ARV) of HbA1c and FPG were calculated from post-baseline visits through 24 months; at least three HbA1c and FPG measurements were required.

All Cox models adjusted for age, sex, race, education, BMI, diabetes duration, randomized treatment arm(s), baseline CVD, heart failure, depression, smoking, alcohol use, baseline HbA1c, and baseline FPG.

Attenuation was calculated as the percentage reduction in the living-alone log-HR after adding the glycemic mediator; negative values indicate no attenuation.

For readability, this table is displayed in long format. For all-metrics models, the same living-alone HR, P value, and attenuation estimate are repeated for each glycemic metric included in that model.

## Supplementary Table 10. Exploratory depression attenuation analysis for neuropathy outcomes

| **Section** | **Outcome** | **Model** | **Participants/events** | **Living alone estimate (95% CI)** | **P-value** | **Attenuation in living-alone log-HR** |
| --- | --- | --- | --- | --- | --- | --- |
| Exposure-mediator association | Baseline depression | Adjusted logistic model | 10,247/2,419 | OR 1.47 (1.31, 1.65) | <0.001 |  |
| Exposure-mediator association | Baseline antidepressant use | Adjusted logistic model | 10,249/1,348 | OR 1.19 (1.03, 1.37) | 0.019 |  |
| Neuropathy outcome model | Neuropathy: MNSI score >2.0 | Without depression/antidepressant | 5,625/3,212 | HR 1.17 (1.07, 1.27) | <0.001 |  |
| Neuropathy outcome model | Neuropathy: MNSI score >2.0 | + baseline depression | 5,625/3,212 | HR 1.14 (1.04, 1.24) | 0.004 | 17.1% |
| Neuropathy outcome model | Neuropathy: MNSI score >2.0 | + baseline antidepressant use | 5,625/3,212 | HR 1.16 (1.06, 1.26) | 0.001 | 5.7% |
| Neuropathy outcome model | Neuropathy: MNSI score >2.0 | + depression and antidepressant use | 5,625/3,212 | HR 1.14 (1.04, 1.24) | 0.004 | 17.1% |
| Neuropathy outcome model | Neuropathy: ankle jerk loss | Without depression/antidepressant | 6,582/3,125 | HR 1.13 (1.03, 1.23) | 0.007 |  |
| Neuropathy outcome model | Neuropathy: ankle jerk loss | + baseline depression | 6,582/3,125 | HR 1.11 (1.02, 1.21) | 0.020 | 13.6% |
| Neuropathy outcome model | Neuropathy: ankle jerk loss | + baseline antidepressant use | 6,582/3,125 | HR 1.13 (1.03, 1.23) | 0.008 | 1.1% |
| Neuropathy outcome model | Neuropathy: ankle jerk loss | + depression and antidepressant use | 6,582/3,125 | HR 1.11 (1.02, 1.21) | 0.021 | 14.3% |

Note. Baseline depression was coded from the ACCORD baseline depression indicator; baseline antidepressant use was derived from medication records at visit 0.

Exposure-mediator models were adjusted logistic models for the association of living alone with baseline depression or antidepressant use.

Neuropathy outcome models were Cox models adjusted for age, sex, race, education, BMI, diabetes duration, randomized treatment arm(s), baseline CVD, heart failure, smoking, and alcohol use.

Attenuation was calculated as the percentage reduction in the living-alone log-HR after adding depression and/or antidepressant use.

## Supplementary Table 11. Benjamini-Hochberg false-discovery-rate sensitivity using raw model P values

| **Outcome family** | **Test** | **Raw P value** | **Family-specific BH q value** | **Global BH q value** |
| --- | --- | --- | --- | --- |
| Longitudinal metabolic levels | HbA1c | 0.001 | 0.005 | 0.005 |
| Longitudinal metabolic levels | FPG | 0.004 | 0.012 | 0.021 |
| Longitudinal metabolic levels | SBP | 0.309 | 0.309 | 0.501 |
| Longitudinal metabolic levels | DBP | 0.027 | 0.040 | 0.070 |
| Longitudinal metabolic levels | LDL | 0.009 | 0.017 | 0.037 |
| Longitudinal metabolic levels | HDL | 0.263 | 0.309 | 0.470 |
| Metabolic variability | HbA1c SD | 0.019 | 0.035 | 0.058 |
| Metabolic variability | HbA1c ARV | 0.059 | 0.089 | 0.144 |
| Metabolic variability | FPG SD | 0.226 | 0.301 | 0.426 |
| Metabolic variability | FPG ARV | 0.513 | 0.616 | 0.623 |
| Metabolic variability | SBP SD | <0.001 | <0.001 | <0.001 |
| Metabolic variability | SBP ARV | <0.001 | <0.001 | <0.001 |
| Metabolic variability | DBP SD | 0.001 | 0.002 | 0.005 |
| Metabolic variability | DBP ARV | <0.001 | 0.002 | 0.005 |
| Metabolic variability | LDL SD | 0.889 | 0.889 | 0.889 |
| Metabolic variability | LDL ARV | 0.769 | 0.838 | 0.792 |
| Metabolic variability | HDL SD | 0.020 | 0.035 | 0.058 |
| Metabolic variability | HDL ARV | 0.010 | 0.025 | 0.039 |
| Exploratory microvascular/neuropathy outcomes | UKPDS composite | 0.440 | 0.640 | 0.576 |
| Exploratory microvascular/neuropathy outcomes | UKPDS + neuropathy composite | 0.324 | 0.639 | 0.501 |
| Exploratory microvascular/neuropathy outcomes | SCr doubling/eGFR decrease | 0.564 | 0.695 | 0.662 |
| Exploratory microvascular/neuropathy outcomes | Macroalbuminuria | 0.210 | 0.639 | 0.420 |
| Exploratory microvascular/neuropathy outcomes | Renal failure/ESRD/SCr >3.3 | 0.750 | 0.750 | 0.792 |
| Exploratory microvascular/neuropathy outcomes | Any nephropathy outcome | 0.628 | 0.707 | 0.711 |
| Exploratory microvascular/neuropathy outcomes | Microalbuminuria | 0.192 | 0.639 | 0.408 |
| Exploratory microvascular/neuropathy outcomes | Photocoagulation/vitrectomy | 0.399 | 0.639 | 0.543 |
| Exploratory microvascular/neuropathy outcomes | Cataract extraction | 0.372 | 0.639 | 0.527 |
| Exploratory microvascular/neuropathy outcomes | 3-line visual acuity decline | 0.312 | 0.639 | 0.501 |
| Exploratory microvascular/neuropathy outcomes | 3-line visual acuity decline using VAS | 0.165 | 0.639 | 0.373 |
| Exploratory microvascular/neuropathy outcomes | Severe vision loss | 0.663 | 0.707 | 0.727 |
| Exploratory microvascular/neuropathy outcomes | MNSI score >2.0 | 0.004 | 0.068 | 0.021 |
| Exploratory microvascular/neuropathy outcomes | Vibratory sensation loss | 0.496 | 0.661 | 0.623 |
| Exploratory microvascular/neuropathy outcomes | Ankle jerk loss | 0.020 | 0.162 | 0.058 |
| Exploratory microvascular/neuropathy outcomes | Pressure sensation loss | 0.368 | 0.639 | 0.527 |

Note. Family-specific BH q values were computed within each outcome family. Global BH q values were computed across all rows as a stricter exploratory sensitivity check.

# Supplementary Figures

## Supplementary Figure 1. Conceptual framework

Conceptual framework for the assumed relation between baseline adult co-residence, measured and unmeasured social or clinical factors, medication routines, cardiometabolic stability, and exploratory complications.

## Supplementary Figure 2. Forest plot of fully adjusted model estimates.

Note. Points represent fully adjusted Model 3 beta estimates for the association between living alone and each metabolic indicator; horizontal bars indicate 95% confidence intervals. Positive values indicate higher levels among participants living alone. Outcomes are shown in their original units, so effect sizes should be interpreted within each metabolic domain rather than compared directly across domains.

## Supplementary Figure 3. RCT subgroup longitudinal trajectories.

Note. Lines show mean values over follow-up stratified by living-alone status and randomized treatment/control assignment within the relevant ACCORD domain. Shaded bands indicate 95% confidence intervals. Panels show HbA1c, fasting plasma glucose, systolic blood pressure, diastolic blood pressure, LDL cholesterol, and HDL cholesterol. The figure is intended to display whether the living-alone pattern was broadly similar within randomized treatment subgroups; formal treatment-interaction testing was not the primary aim of this analysis.
